# Assessing the health outcomes, productivity gains, and budget impact of anti-PD-(L)1’s in the early stages of cancer treatment in Greece

**DOI:** 10.3389/fonc.2026.1814460

**Published:** 2026-08-19

**Authors:** Nikolaos Themistoklis Yfantopoulos, Panagiota Naoum, Anastasios Skroumpelos, Antonis Karokis, Demet Sönmez, Raquel Aguiar-Ibañez, Vivek Khurana, Anastasios Boutis, Michail Liontos, Zacharenia Saridaki, Kostas Athanasakis

**Affiliations:** 1 MSD Greece, Athens, Greece; 2 Institute for Health Economics, Athens, Greece; 3 Value & Implementation Global Medical and Scientific Affairs, MSD, Stockholm, Sweden; 4 Value & Implementation Outcomes Research, Merck & Co., Inc., Rahway, NJ, United States; 5 Value & Implementation Outcomes Research, MSD Sharp & Dohme GmbH, Munich, Germany; 6 3rd Department of Medical Oncology, Theagenio Cancer Hospital, Thessaloniki, Greece; 7 Oncology Unit, Department of Clinical Therapeutics, Medical School and National and Kapodistrian University of Athens, Alexandra Hospital, Athens, Greece; 8 1st Oncological Clinic, Metropolitan Hospital, Athens, Greece and Oncology Department, “Asklepios DIAGNOSIS”, Heraklion, Greece; 9 Department of Public Health Policy, University of West Attica, Athens, Greece

**Keywords:** access and affordability, anti_ PD_1, cancer innovation, early stage, Greece, immunotherapy, melanoma, PD-(L)1 inhibitors

## Abstract

**Objectives:**

Cancer incidence and mortality have been increasing both globally and in Greece. The aim of this study was to quantify the health benefits and budget impact of including anti-PD-(L)1’s in three early-stage cancer indications, adjuvant melanoma stage IIB/C and III, adjuvant renal cell carcinoma, and perioperative triple-negative breast cancer.

**Methods:**

A Markov model approach was used to predict clinical outcomes and costs throughout the average patient pathway over a 10-year time horizon. The model includes two scenarios: (1) using anti-PD-(L)1’s for patients with early-stage and metastatic disease (ESD scenario) versus (2) treatment with the previous standard of care in the early-stage and anti-PD-(L)1’s only in the metastatic disease setting (reference scenario). Estimated outcomes include life-years (LYs), quality-adjusted life-years (QALYs), events or recurrences, productive LYs lost for patients and caregivers, active treatments administered for metastatic disease, deaths, and costs. The model inputs included efficacy data from clinical trials, Greek data on market shares, projected eligible patients, and the respective costs.

**Results:**

Results indicate the following in the ESD scenario: a reduction of 1,722 (25%) recurrences, 881 (24%) deaths, an increase of 5,555 (11%) LYs without recurrence, an increase of productive LYs by 901 (16%) and 2,363 QALYs gained (4%), and a decrease of 1,782 (27%) in active treatments for the metastatic disease setting. The average incremental budget impact per patient per annum was €31,853 throughout the 10-year time horizon of the model from 2023 to 2032.

**Conclusion:**

Results indicate that adjuvant/perioperative treatments could provide a significant benefit to patients with cancer, with a manageable additional cost for the healthcare system. The estimations of the model demonstrate a strong call to action for policymakers to enhance access to innovative treatments, since benefits arise for patients, their caregivers, and society as a whole through increased productivity gains.

## 1 Background

According to the World Health Organization (WHO), one out of five adults is expected to develop cancer in their lives. Moreover, the incidence of cancer is also steadily increasing (1). Hence, the European Union (EU) has developed a comprehensive framework, EU beating cancer plan, to prepare and address the increasing cancer care needs (2). The burden of disease in Greece is also high with an estimated 64,530 new cancer cases and 33,166 deaths annually (3). This burden is expected to rise substantially, with cancer incidence projected to increase by 16.5% and mortality anticipated to rise by 23.7% by 2045 (4). A study was conducted to clarify and provide a hierarchical order of the local healthcare priorities, in which cancer was underlined as the leading priority, using the MCDA (Multi-Criteria-Decision-Analysis) methodology (5).

The latest trend in cancer management is shifting the current paradigm, in alignment with the EU beating cancer plan, towards prevention and treatment at earlier disease stages, offering patients improved health outcomes, and potentially even cure (6). Immune checkpoint inhibitors have been established as a backbone therapy in the metastatic setting by prolonging both overall survival (7–9) and progression-free survival (10–12) in comparison to the previous standard of care.

In addition, immunotherapies have demonstrated significant results in clinical trials in the early-stage setting, by prolonging event/disease-free survival (13–15), which translates into significant overall survival benefits (16–18). The KEYNOTE 564 trial in renal cell carcinoma (RCC), the KEYNOTE 522 trial in triple-negative breast cancer (TNBC), and the KEYNOTE 671 trial in non-small cell lung cancer (NSCLC) have all demonstrated a statistically significant and clinically meaningful overall survival benefit in favor of the pembrolizumab regimen, with a hazard ratio (HR) of 0.62 (95% CI: 0.44−0.87) compared to the previous standard of care in RCC, an HR of 0.65 (95% CI: 0.51−0.85) in the pembrolizumab–chemotherapy group compared to the placebo–chemotherapy group in TNBC, and an HR of 0.72 (95% CI: 0.56−0.93) compared to placebo in NSCLC (16–18). Therefore, this paradigm shift in the early stage appears to be promising for patients with cancer with potential benefits that could extend to the entire healthcare system and society.

In this context, this study aims to estimate (1) the health outcomes, (2) healthcare expenditures, and (3) patient’s and caregiver’s productivity gains of three types of cancers: melanoma stage IIB/C and III, RCC, and TNBC. The scenarios tested in this study are: initiating anti-PD-(L)1 treatments in the early-stage setting, while also including them in the metastatic disease setting (ESD scenario) versus reserving anti-PD-(L)1’s only for metastatic disease treatment, and using the previous standard of care in the early-stage setting (reference scenario), in Greece, from 2023 to 2032. The structure of the model, used to test these scenarios is shown in **Appendix B**.

## 2 Materials and methods

**Figure 1:**
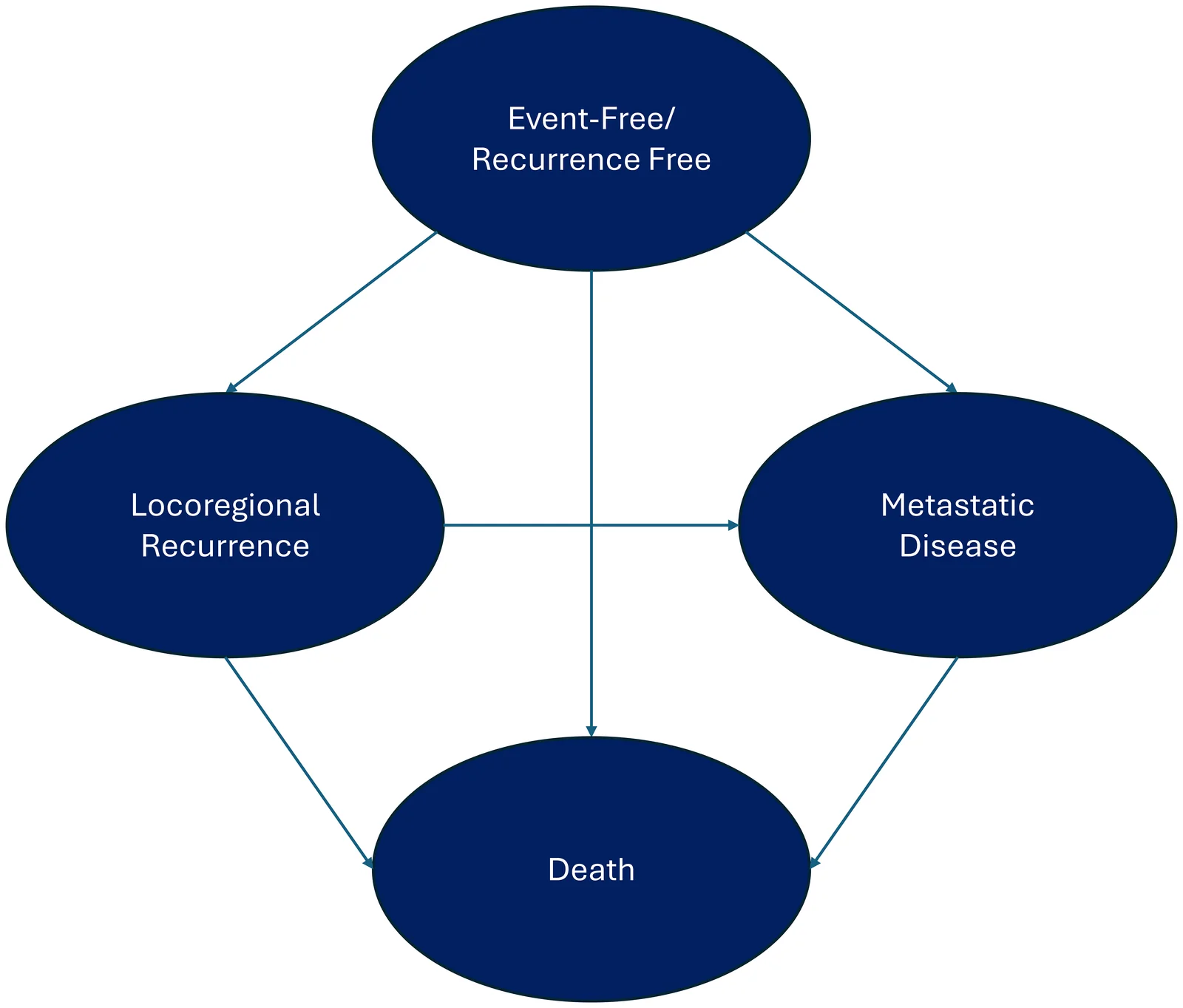
Markov model health transition states.

A Markov model approach (**Figure 1**) was used to estimate health outcomes, costs, and productivity gains of using anti-PD-(L)1 agents in the treatment of three early-stage cancers—stage IIB/C and stage III melanoma stage following completion resection, RCC at increased risk of recurrence following nephrectomy or following nephrectomy with resection of metastatic lesions, and TNBC with locally advanced or early-stage cancer at high risk of recurrence—over a 10-year time horizon from 2023 to 2032. The immunotherapies examined were pembrolizumab and nivolumab for stage IIB/IIC and stage III melanoma, and pembrolizumab as the only approved immunotherapy for the adjuvant treatment of patients with RCC and for the perioperative treatment of high-risk patients with TNBC. The official EMA labels of the three early-stage indications are presented in **Appendix A**. Costs and outcomes were discounted by 3% annually—as in the relevant HTA submissions. An alternative discounting scenario is presented in **Appendix G**.

The model’s structure is based on the independent cost-effectiveness models (CEMs) of each indication. These have been evaluated and reviewed, and each indication has been reimbursed in various healthcare systems across Europe, including Greece (19–22), France (23–25), Germany (26–28), Italy (29–31), and the United Kingdom (32–35). The model compared two scenarios: (1) a world where anti-PD-(L)1 treatments are available both in the early and the metastatic disease setting (ESD scenario) and (2) a world where anti-PD-(L)1 treatments are available exclusively in the metastatic setting, with the previous standard of care administered in the early stage (reference scenario). Our study compares these two scenarios over a 10-year period from 2023 to 2032. The structure of the model can be found in **Appendix B** (3).

In more detail, the model examined health outcomes and costs from the time that patients initiate perioperative or adjuvant treatment. The model uses weekly cycles, with half-cycle correction applied. Health outcomes included life-years (LYs) gained, quality-adjusted life-years (QALYs) gained, total recurrences, deaths, and productive LYs lost for patients and caregivers. All costs included in the model reflected the real-world treatment setting of cancer care from the initiation of early-stage treatment to death. These included drug acquisition cost, drug administration cost, disease management cost, surgery cost, adverse event management cost, terminal care, and productivity cost for patients and caregivers.

The latest available GLOBOCAN data were utilized to identify the incidence of each cancer type in Greece (36). Thereafter, the eligible patient population was calculated based on an epidemiological breakdown of the Greek population by cancer stage—due to the absence of a national cancer registry. The base case inputs and assumptions of the model can be found in the Table in **Appendix C**. The analysis was conducted adopting a societal perspective as both direct and indirect costs were taken into account.

The population growth rate was calculated based on two available published time points for the Greek population. An interpolation analysis was conducted to calculate the percentage growth in the population from 2011 to 2021 (37), and the annual (negative) percentage growth was applied from 2021 onwards. According to the literature, the Greek clinical practice, and the use of immunotherapies in relevant clinical trials, patients were eligible to be re-treated with anti-PD-(L)1’s if they completed the anti-PD-(L)1 treatment course (1 year) and then experienced a recurrence at least 6 months after completing the treatment course. A recent study utilizing the methodology of a Delphi panel with clinicians from 10 countries, including France, Germany, Spain, and Italy, has also substantiated that re-treatment within the time frame of 3–6 months after recurrence is preferable for clinicians (38). The treatment’s market shares in the metastatic stage were conditional to the adjuvant and perioperative market shares and were calculated based on the latest available local market research data.

### 2.1 Efficacy

The transition probabilities within the health states of the model were based on the CEM analyses that have been reviewed and accepted by HTA agencies such as the National Institute for Health and Care Excellence (NICE) (32–35), as well as the Greek HTA committee. The parametric extrapolations and goodness-of-fit assessments were inherited from the underlying HTA-accepted CEMs and were not re-evaluated in the present study to maintain consistency. Additionally, all these indications have been found to be cost-effective and are fully reimbursed in Greece (19–22). As in the CEMs, parametric distributions were fitted to patient-level data to estimate the transition probabilities of patients within the different health states. For more details on how transition probabilities were calculated, please refer to **Appendix D**.

### 2.2 Utilities

Utilities for each health state and disutilities for each adverse event, by treatment setting and treatment line, were incorporated in the model. Health state utilities were derived from the relevant clinical trials (39–41) and the European algorithm of the EQ-5D instrument was used to map the utilities (42, 43). Adverse event disutilities were applied as one-off events at treatment initiation. The Ara and Brazier (2010) (44) model of mean utility values was used to adjust utility values according to age and sex.

### 2.3 Cost inputs

The drug acquisition cost was calculated using invoice prices, which include state mandatory discounts (−13.3%) applied on the ex-factory price of each product (45). Ex-factory prices were derived from the latest available published price bulletin (46). The costs for drug administration were also retrieved from the relevant legislation, while vial sharing was not considered since it is not practiced in Greece (46). The costs for end-of-life care were assumed to be similar for all indications and were based on the only available Greek study conducted on terminal care costs for lung cancer (47). The disease management and adverse event costs were derived from the Greek Diagnosis Related Groups (DRG) system (48). Costs related to physician visit, oncology nurse visits, and outpatient visits were obtained from Greek studies that included this information (49, 50). Indirect costs were estimated with data from Eurostat, and based on the current legislation (51). The hours of productive work lost from absenteeism and presentism for both patients and their caregivers were drawn from the literature due to the lack of availability of Greek data on this subject (52). The price year for the estimation of costs was 2023.

### 2.4 Model validation

The model’s structure and efficacy inputs have been reviewed and accepted by NICE, as mentioned in the efficacy section above. A thorough process of external reviews and presentations to key stakeholders has been followed to ensure the external validity of our results. The inputs used have been reviewed and approved by local experts who specialize in cost-effectiveness analyses and assessments and from the Institute of Health Economics (i-hecon) in Greece.

## 3 Results

Overall, the introduction of anti-PD-(L)1 agents in early-stage disease (ESD) is expected to yield the following: 5,555 additional LYs in the recurrence-free state (+11%), 2,556 additional LYs (+4%), and 2,363 additional QALYs (+4%). Moreover, a 14% decrease is demonstrated in productive hours lost for patients and a 22% decrease in productive hours lost for caregivers, which translate to a total averted loss of 901 (16%) productive LYs for patients and caregivers.

Additionally, a 25% decrease is shown in prevented recurrences. Most importantly, 881 deaths (−24%) and 934 deaths after the first recurrence (−30%) are expected to be averted. Lastly, 1,782 active treatments in the metastatic disease stage (−27%) are expected to be prevented with the addition of anti-PD-(L)1 treatments in the early-stage setting over a 10-year time horizon. These data are presented in **Table 1**. The disaggregated results per indication are presented in **Appendix H**.

**Table 1:** Health outcomes over a 10-year period.

Outcomes	Reference scenario	ESD scenario	Difference	Percentage change
Life years	64,330	66,886	2,556	+4%
Life years—recurrence free	50,876	56,431	5,555	+11%
QALYs	53,844	56,207	2,363	+4%
Events or recurrences	6,887	5,165	−1,722	−25%
Patients receiving active treatment for metastatic disease	6,725	4,943	−1,782	−27%
Deaths	3,613	2,732	−881	−24%
Productive years lost for patients	5,543	4,747	−796	−14%
Productive years lost for caregivers	479	374	−105	−22%
Productive years lost for patients and caregivers	6,022	5,121	−901	−16%

**Figure 2:**
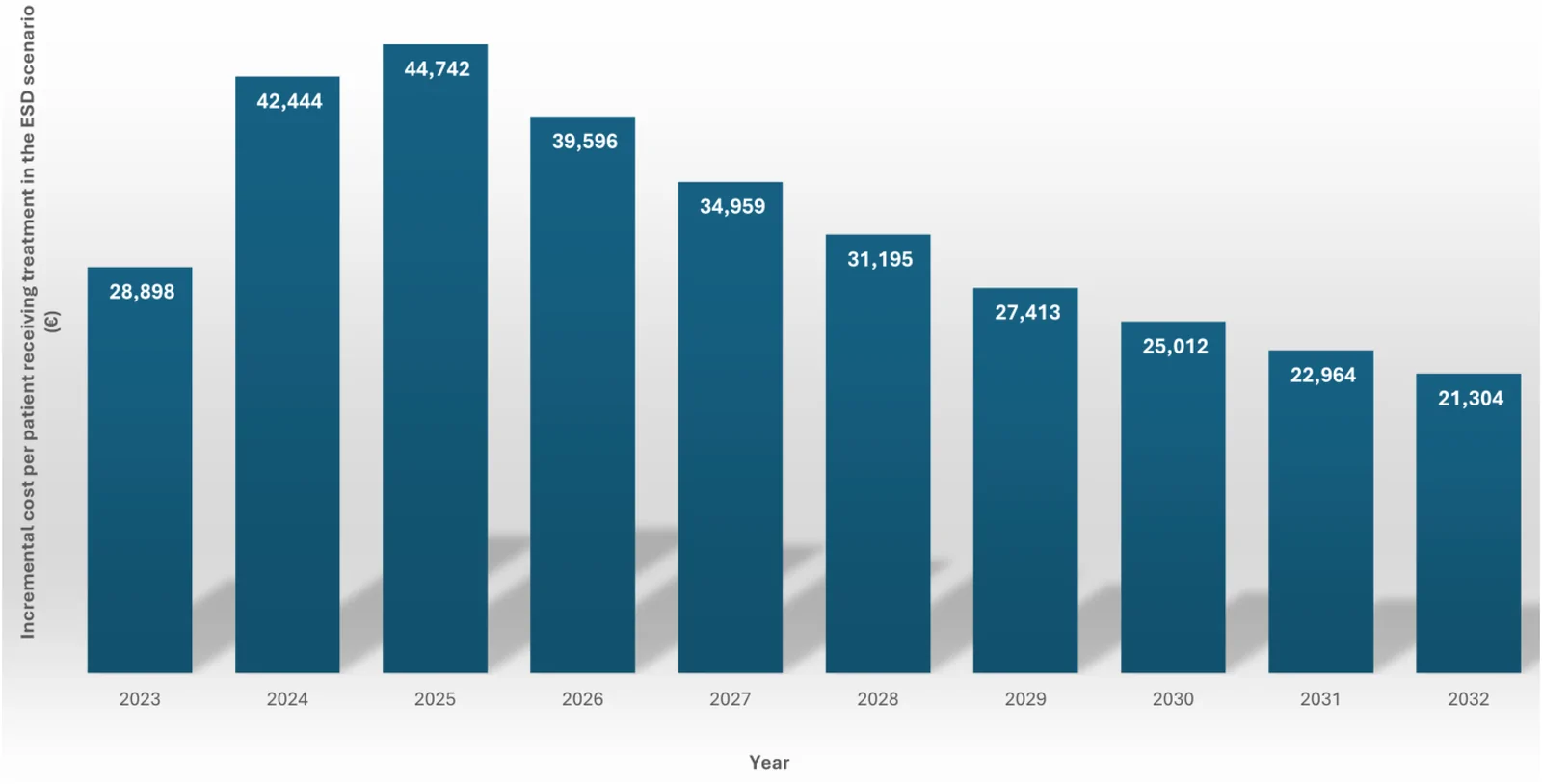
Annual incremental budget impact per patient receiving treatment in the ESD scenario compared to the reference scenario.

The healthcare cost analysis is shown in **Figure 2**. The incremental annual average cost per patient receiving treatment with anti-PD-L1 agents from 2023 to 2032 is estimated at €31,853. The main drivers of the incremental cost in the ESD scenario were administration and treatment acquisition costs. It is noteworthy that the incremental costs of anti-PD-(L)1’s increase from 2023 to 2025, where it reaches a peak, and thereafter steadily decreases until the last year of the analysis—2032. The incremental cost per patient showed a decline from 2026 to year 2032 of 52%, which was mainly attributed to cost offsets presented in **Table 2**. The primary sources of cost offsets stem from the prevention of metastatic disease treatment costs, followed by reduced productivity losses, disease management costs, and decreased terminal care costs. The increasing cost offsets are graphically in **Appendix F**. The disaggregated economic results per indication are presented in **Appendix I**.

**Table 2:** Annual incremental budget impact per patient receiving treatment in the ESD scenario compared to the reference scenario, per treatment category.

Year	Early stage(€)	Salvage surgery(€)	Disease manage ment (€)	1L Metastatic (€)	2L Metastatic (€)	Adverse events (€)	Termina l care (€)	Productivity losses (€)	Net budget impact (€)
2023	29,522	−7	−38	−385	−73	48	−7	−162	28,898
2024	46,138	−16	−131	−2,050	−539	61	−60	−960	42,444
2025	53,446	−23	−265	−4,324	−1,401	56	−187	−2,560	44,742
2026	53,448	−25	−406	−6,288	−2,309	51	−346	−4,528	39,596
2027	53,352	−26	−520	−7,790	−3,080	47	−486	−6,537	34,959
2028	54,037	−27	−615	−9,127	−3,770	42	−617	−8,728	31,195
2029	53,309	−27	−659	−9,832	−4,164	39	−703	−10,550	27,413
2030	53,889	−27	−691	−10,480	−4,493	37	−782	−12,440	25,012
2031	53,879	−28	−699	−10,872	−4,686	34	−841	−13,823	22,964
2032	54,524	−28	−710	−11,398	−4,920	33	−909	−15,288	21,304

Early stage refers to cost in the perioperative and adjuvant setting. The drug acquisition cost and drug administration cost are included in this category. 1L Metastatic and 2L Metastatic categories also account for both drug acquisition and drug administration cost. Productivity costs are reported for both patients and caregivers.

Sensitivity analyses were conducted to identify the parameters that most significantly impact the model’s key outcomes. The parameter with the most significant impact was the uptake of anti-PD-(L)1’s, which, when increased to 100%, could result in up to 1,177 deaths (−34%) prevented (shown in **Appendix E**) and a 21% cost increase, compared to the base case, whereas delaying the launch of anti-PD-(L)1 agents could result in a 9% decrease in both cost and deaths averted. These data are presented in **Table 3**. The impact of these scenario analysis to all parameters examined in the model is presented in **Appendix E**. Additionally, a sensitivity analysis with 0% and 5% discounting is presented in **Appendix G**.

**Table 3:** Sensitivity analysis on the impact of anti-PD-(L)1 agents in the ESD scenario on costs, deaths, and recurrences averted over a 10-year time horizon.

Scenario tested	Percentage change from base case		
Base scenario	Scenario analysis on deaths averted	Scenario analysis on cost	Scenario analysis on recurrences averted
100% uptake of anti-PD-1/PD-L1 agents in future environment	+34%	+21%	+22%
20% increase of target population for all years (manual population estimate only)	+20%	+20%	+17%
20% decrease of target population for all years (manual population estimate only)	−4%	−4%	−4%
Delayed time to launch of anti-PD-1/PD-L1 agents in future environment	−9%	−9%	−9%
Do not account for relative dose intensity	No change	−3%	No change

## 4 Discussion

The aim of this study was to estimate the health benefits of adding innovative immunotherapies in the early stage setting for three indications (melanoma, RCC, and TNBC) while also evaluating the costs associated with them. It is important to note that early-stage treatments for these indications are reimbursed in Greece and recommended in the local therapeutic protocols produced by HesMO (Hellenic Society of Medical Oncology) (53). The indications included in the model are especially important for the Greek healthcare environment, because of their severity, in terms of incidence and mortality. More specifically, the incidence of melanoma is expected to increase by more than 50% from 2020 to 2040 (54). Melanoma-attributed mortality is also showing a sharp increase and is expected to reach 96,000 annual deaths by 2040— an increase of 69% compared to 2020 (54). Kidney cancer ranks 10th in terms of incidence and 11th in terms of mortality in Greece while clear cell RCC is the most common form of kidney cancer, accounting for 9 out of 10 cases. Lastly, breast cancer has the second highest incidence and third highest mortality in Greece (3). TNBC is the most aggressive subtype of breast cancer (constituting around 15%–20% of all breast cancers), demonstrating the highest mortality rate among all other subtypes of breast cancer, in both the early and metastatic setting (55, 56).

Our study showed that the addition of anti-PD-(L)1 agents in the ESD scenario is estimated to contribute 5,555 additional LYs gained in the recurrence-free state (+11%), 1,722 less recurrences (−25%), and 881 less deaths (−24%), compared to the reference scenario, from 2023 to 2032. These results demonstrate that the addition of anti-PD-(L)1 agents in the early-stage treatment setting has the potential to improve the life of patients with cancer. The potential of long-term survival without recurrence, is important for patients with cancer and their families. Moreover, the incremental average cost is projected at €31,853 from 2023 to 2032, and as low as €21,304 in the final year of the analysis.

According to the latest update of market research data and projections made, 78% of all eligible patients are projected to receive early-stage treatment in our analysis (presented in **Appendix J**). In a discrete choice experiment (DCE) about adjuvant melanoma treatment, patients chose to receive active treatment in 78% of their choice tasks—with physicians choosing to administer active treatment in 79% of the choice tasks—in line with the results of our analysis (57). Another interesting finding from this DCE is that 10-year survival was the patient’s first preference, followed by avoiding fatigue and achieving 3-year recurrence-free survival (57). Patients mention fear of recurrence as one of the most common issues reported regarding their mental health. As such, it is logical that a sustained period of recurrence-free survival is particularly important for them (58). This further substantiates the importance of the findings of the model, which demonstrate a significant increase in LYs without recurrence of 11% (5,555 additional LYs without recurrence) and 16% of productive LYs for both patients and caregivers (901 productive LYs gained for patients and caregivers combined).

As outlined earlier, patients value a sizeable increase in their overall survival the most. However, an overall survival benefit, especially in the early stage, might take several years to prove (59). Denying patients access to innovative treatments for such long periods poses a significant ethical dilemma. Additionally, a statistically meaningful correlation between RFS HR and OS HR has been indicated in melanoma (60), while several other studies in the literature have demonstrated a positive association between DFS, EFS, and OS in several indications including TNBC, RCC, and lung cancer (61–63). Indicatively, the KEYNOTE 564 clinical trial in RCC, the KEYNOTE 522 clinical trial in TNBC, and the KEYNOTE 671 trial in NSCLC have all demonstrated a statistically significant overall survival benefit, which highlights this association (16–18). As a result, investing in treating patient early has the potential to provide important benefits in terms of overall survival for patients, with as many as 881 deaths (24% decrease) projected to be averted by 2032.

The findings of this study show consistency with the direction of similar studies across different countries, including Belgium, Switzerland, Czech Republic, Hungary, and the United Kingdom (64–69). However, the generalizability of these results is limited due to differences in the gene pool of the population, clinical practice, screening programs, early-stage diagnosis, and treatment patterns across the EU.

This is the first study to assess the impact of anti-PD-(L)1 in the early-stage setting on both costs and outcomes. Similar studies have been presented for Belgium and Switzerland, but their focus was mainly on clinical outcomes. The results are similar across all studies, with the Belgian study showing a 25% decrease in recurrences, 29% of metastatic disease treatments avoided, and 25% decrease in deaths, and the Swiss study showing a 27% decrease in recurrences, 35% of metastatic disease treatments avoided, and a 23% decrease in deaths (64, 65).

Lastly, two more recent publications have reported on productive LYs gained in England and Scotland by lowering presentism and absenteeism for patients and caregivers. In England, a 17% increase in productive LYs was predicted with overall LYs and QALYs projected to be increased by 3% (66), whereas in Scotland, the predicted increase in productive LYs was 16%, with the same reported increase in LYs and QALYs (67). In our study, the magnitude of the predicted increase was 16% in the incremental productive LYs and 4% increase in the incremental QALYs gained, which is similar to the studies listed above.

This study aims to provide healthcare systems with informative data on the overall benefit of immunotherapies in the perioperative and adjuvant setting. The model’s projections are conservative, considering both the methods used for parametric extrapolations, which have been assessed and reimbursed by multiple HTA agencies across the EU (Greece (19–22), France (23–25), Germany (26–28), Italy (29–31), and UK (32–35)).

Some of the limitations of the model include that, within the 10-year time horizon, patients who receive early-stage treatment with immunotherapy in the final years of the model’s cohort, particularly in the last 3 years, are administered treatment and the cost is included in the model. However, the incremental outcomes for these patients will present beyond the model’s time horizon, as shown by the increasing health benefits and productivity gains from year 3 to year 10 (presented graphically in **Appendix F**). Hence, the outcomes presented for patients are underestimated due to the 10-year time horizon, instead of the full lifetime of a patient. As such, utilizing a cohort of patients and following them throughout their lifetime would have perhaps been a more appropriate approach and an idea that could be further explored in future research. Another limitation of the model concerns the cost analysis of the model. The cost analysis was conducted using invoice prices (ex-factory price −13.3%) included in the mandatory legislated discounts for all hospital drugs in Greece (45). These prices do not account for the volume rebate and clawback attributed to each drug in Greece, as well as the confidential discounts provided in the formal reimbursement process. Thus, the incremental budget impact of the anti-PD-(L)1 agents in the early stage (€31,853 annually on average per patient) is overestimated. Despite this, the overall additional cost of introducing anti-PD-(L)1 agents into the early-stage setting appears to be manageable compared to the benefits these treatments offer.

Productivity loss inputs were derived from a US-based survey because Greek-specific data for early-stage cancers are not currently available. Differences between Greece and the United States—such as employment protections, paid sick leave, and caregiver roles—may affect the absolute estimates of productivity loss. However, because the model compares two scenarios using the same inputs, any systematic bias would likely affect both equally and is therefore unlikely to materially change the relative differences or the overall direction of the findings. Future research using Greek-specific productivity data could further refine these estimates.

A further limitation is that the model relies on clinical trial data and epidemiological estimates, which may not fully capture real-world variation in patient characteristics, treatment adherence, and healthcare delivery. However, because the analysis is intended to compare relative differences between scenarios rather than estimate absolutes, this uncertainty is unlikely to change the overall direction of the productivity benefit. Future studies using Greek real-world evidence would help validate and refine these estimates.

The use of pembrolizumab as a proxy for the efficacy of the PD-1 inhibitor class should also be interpreted with caution. In the absence of more detailed data, the efficacy inputs were retrieved from the pembrolizumab studies (70–72). Future work should be implemented to test this assumption. Local real-world evidence studies could be used to examine treatment effectiveness of the available therapeutic alternatives in the real-world setting. To conclude, the consistent direction of the results from multiple countries demonstrates the benefit of immunotherapy treatments for early-stage patients, although the magnitude of this benefit needs to further be explored.

## 5 Conclusions

Long-term survival and better quality of life are two of the key priorities for patients with cancer. Hence, treating patients in the early-stage setting with anti-PD-(L)1’s, rather than waiting to treat patients after they have developed metastatic disease, could be beneficial. The potential of avoiding 1,722 recurrences and as many as 881 total deaths for an average incremental cost of €31,853 per patient within 10 years is a very important prospect for patients, caregivers, and society as a whole. In turn, this leads to significant productivity gains for cancer survivors. Our analysis showed that early-stage treatment could prevent almost 1,800 metastatic disease treatments and decrease productivity losses (for patients and caregivers) by 16%; thus contributing to the Greek economy.

It can be concluded that adjuvant and/or perioperative anti-PD-(L)1 treatments in Greece appear to demonstrate significant improvement in health outcomes and productivity gains. Thus, they have been established as the new standard of care for patients with early-stage cancer in Greece.

## Data Availability

The original contributions presented in the study are included in the article/supplementary material. Further inquiries can be directed to the corresponding author.
