# Supplementary material for: Assessing the health outcomes, productivity gains, and budget impact of anti-PD-(L)1’s in the early stages of cancer treatment in Greece
Source: Front Oncol. 2026 Aug 19;16:1814460. doi: 10.3389/fonc.2026.1814460 (PMC13535520; doi:10.3389/fonc.2026.1814460)
Supplement: Supplementary file 1 [file Supplementaryfile1.docx]

# 6 Appendices

## Appendix A

The early-stage indications of patients eligible to receive IO treatment considered in the model, are presented underneath:

|  | Tumor | Therapeutic indication in EMA Label |
| --- | --- | --- |
| Pembrolizumab | Melanoma | Adjuvant Treatment of adults with Stage IIB, IIC or Stage III melanoma and who have undergone complete resection.[67] |
|  | Triple Negative Breast Cancer | Adults with locally advanced, or early-stage triple-negative breast cancer (TNBC) at high risk of recurrence. IO is administered in combination with chemotherapy as perioperative treatment, and then continued as monotherapy as adjuvant treatment after surgery. [68] |
|  | Renal Cell Carcinoma | Adjuvant treatment of adults with renal cell carcinoma at increased risk of recurrence following nephrectomy or following nephrectomy and resection of metastatic lesions. [69] |

| Nivolumab | Melanoma | Patients eligible for the adjuvant treatment of adults and adolescents 12 years of age and older with stage IIB /IIC or stage III or stage IV melanoma who have undergone complete resection.[70; 71] |
| --- | --- | --- |

### Appendix B

Outcomes	in	the	model	include	life	years	(LYs),	quality-adjusted	life	years	(QALYs), events/recurrences, active treatments for metastatic disease, deaths after recurrence, and productive hours lost per patient and caregiver. The cost associated with each health state and disaggregated cost per category of cancer management including drug acquisition cost, drug administration cost, adverse event cost, disease management cost (healthcare resource use), surgery cost and societal cost( including productive hours lost).

**Model Diagram**

### Appendix C

The base case analysis inputs are presented in the table below:

| **Input** | **Value** |
| --- | --- |
| **Perspective** | Societal |
| **Time horizon** | 10 years |
| **Discounting** | Clinical outcomes & costs: 3% |
| **Indications** | 1. Melanoma Stage III 2. Melanoma Stage IIB/C 3. Renal cell carcinoma at increased risk of recurrence as per the EMA label presented in Appendix A 4. Triple negative breast cancer at increased risk of recurrence as per the EMA label presented in Appendix A |
| Baseline age for each indication | The starting age of the clinical trials have been used, in the absence of a national registry. The detailed starting ages per indication are:  Melanoma stage IIB-C [[37](https://mydrive.merck.com/personal/yfantopo_merck_com/Documents/Desktop/2026%20projects/HOBIP%20Manuscript%20Comments/Hungarian%20HOBIP%20review/Manuscript_Clean_012326.docx#_ENREF_37)]: 59.3 years  Melanoma stage III [[34](https://mydrive.merck.com/personal/yfantopo_merck_com/Documents/Desktop/2026%20projects/HOBIP%20Manuscript%20Comments/Hungarian%20HOBIP%20review/Manuscript_Clean_012326.docx#_ENREF_34)]: 54.0 years  RCC [[35](https://mydrive.merck.com/personal/yfantopo_merck_com/Documents/Desktop/2026%20projects/HOBIP%20Manuscript%20Comments/Hungarian%20HOBIP%20review/Manuscript_Clean_012326.docx#_ENREF_35)]: 67.3 years  TNBC [[36](https://mydrive.merck.com/personal/yfantopo_merck_com/Documents/Desktop/2026%20projects/HOBIP%20Manuscript%20Comments/Hungarian%20HOBIP%20review/Manuscript_Clean_012326.docx#_ENREF_36)]: 49.0 years |
| **Population** | 2021 population: 10,432,481  Population Growth rate of -0.3679% applied to year 2021 and onwards.  Target population for each indication: based on Globocan epidemiological data for Greece[36]. Dendrograms have been developed thereafter to estimate the eligible population of Greece for each indication. These data have been thereafter cross-checked using market research data and validated by experts’ opinion. |
| **Health state transitions** | A four -state Markov model has been used. Transition probabilities are informed by clinical trials, NMA or published research as in the relevant NICE submission[32; 33; 34; 35] |
| **Treatment duration** | Based on clinical trials :  In the adjuvant or perioperative setting for pembrolizumab,  /pembrolizumab + chemotherapy and chemotherapy, base case data derived from the Kaplan–Meier curves for time to treatment discontinuation from the KEYNOTE trials.^[39;^ ^40;^ ^41;^ ^72]^ For the remaining treatments and settings/lines of treatment, data derived from parametric models (fitted to clinical trial data or based on reported median data), or equivalence to recurrence-free/progression-free survival or to duration of other treatments was assumed. |
| **Market shares** | Based on market research |
| **Retreatment with anti-PD-(L)1’s** | Patients were eligible to be retreated if they completed the anti-PD(L)1 treatment course (1 year) and then experienced a recurrence at least 6 months after completing their treatment course The market shares of the metastatic stage were conditional to the adjuvant and perioperative market shares and were calculated based on the latest available market research data. |
| **Adverse Events (AEs)** | AEs included are drug-related, and of Grade 3+ with ≥ 5% incidence in  any treatment arm.  AE-related disutilities are accounted for as one-off events at treatment initiation |
| **Health State Utilities** | Informed from relevant clinical trials using European algorithms. [42; 73]  Age- and sex-related disutilities are also considered. |
| **Productivity** | Productivity estimates were assessed for patients and caregivers from a relevant publication in the US due to the lack of available data in Greece**[51]** |

### Appendix D

### Transition Probabilities

In the recurrence-free disease state: Both Pembrolizumab plus chemotherapy combination and the watchful waiting or chemotherapy arms, parametric distributions were fitted to patient-level data from the KEYNOTE trials,^[39;^ ^40;^ ^41;^ ^72]^ and then used to estimate the transition probabilities to the other health states including locoregional recurrence, metastatic disease, and death states. Importantly, transition probabilities to death were defined as the maximum of the trial-based estimates and the background mortality, whichever was higher among the two options.

For the Disease Free to the other states of the model: Transition probabilities for dabrafenib +trametinib for melanoma, are based on the hazard ratio (HR) of recurrence-free survival against pembrolizumab (with the proportion of the overall hazard attributable to each recurrence-free survival failure type assumed to be the same as in the pembrolizumab arm). For nivolumab, equivalence to pembrolizumab was assumed in the absence of relevant, comparable data. In the locoregional state: For melanoma, transitions to the metastatic state were assumed to be equivalent across adjuvant treatment arms. These transitions were based on survival analyses of individual patient-level data from database studies or were assumed equivalent to pembrolizumab (in the case of other anti-PD-(L)1).

For the metastatic state: several methods are used to derive the transitions for 1L metastatic treatments, ranging from parametric models (derived from trial data in the advanced/metastatic setting for melanoma and RCC, and trial data in the metastatic setting for TNBC) to assuming equivalence between treatments. The parametric models were fitted to patient-level data or based on observed median data, with HRs for the remaining treatments derived	from network meta-analyses when available.

### Appendix E

Scenario Analysis on 100% uptake of anti-PDL1 agents and Delayed time to launch of anti-PD(L)1

agents.

| Clinical outcomes | Impact on clinical outcomes – Base case scenario | 100% uptake of anti-PD-(L)1 agents | Delayed time to launch of anti-PD-(L)1 by12- months |
| --- | --- | --- | --- |
| Life years in the recurrence Free  Health State | 5,555 | 7,493  +35% | 4,793  -16% |
| Life-years | 2,556 | 3,521  +38% | 2097  -18% |
| QALYs | 2,365 | 3,238  +37% | 1,962  -21% |
| Number of events or recurrences | -1,722 | 2,200  +28% | 1,586  -8% |
| Number of active treatments for  metastatic disease | -1,782 | 2,244  +26% | 1,700  -5% |
| Number of deaths | -881 | 1,177  +34% | -776  -12% |
| Number of deaths after first event or  Recurrence | -934 | 1,248  +34% | -820  -15% |
| Cost per patient receiving treatment | 31,853€ | 37,985  +19% | 29,137  -9% |
| Averted  Productive Life Years Lost Patients | 796 | 1,256  +45% | 727  -9% |
| Averted  Productive Life Years Lost Caregivers | 104 | 141  +30% | 93  -12% |

### Appendix F

Graphical demonstration of cost offsets per patient receiving treatment, from 2023-2032.

*The societal costs refer to averted productivity losses for patients and caregivers.

### Appendix G

Sensitivity analyses of 0% and 5% discounting in key outcomes categories.

| **Clinical outcomes** | **ESD**  **Base case scenario** | **0% Discounting** | **5 % Discounting** |
| --- | --- | --- | --- |
| **Life years in the recurrence Free**  **Health State** | 5,555 | 6,304  +12.6% | 5,128  -8% |
| **Life-years** | 2,556 | 2,982  +15.4% | 2,316  -9.9% |
| **QALY’s** | 2,365 | 2,746  +14.9% | 2,149  -9.6% |
| **Number of events or recurrences** | -1,722 | 1,827  +5.9% | 1,659  -3.7% |
| **Number of active treatments for**  **metastatic disease** | -1,782 | 1,902  +6.5% | 1,711  -4.1% |
| **Number of deaths** | -881 | 978  +10.4% | -824  -6.7% |
| **Cost per patient receiving treatment** | 31,853€ | 28,899 €  -9.3% | 32,626€  +2.4% |
| **Averted**  **Productive Life Years Lost**  **For Patients** | 796 | 904  +12.7% | 735  -7.9% |
| **Averted**  **Productive Life Years Lost**  **for Caregivers** | 104 | 116  +9.6% | 98  -6.9% |

### Appendix H

Discounted health and productivity outcomes after early-stage disease treatment with anti-PD-(L)1 agents in the Greece from 2023-2032, by cancer type.

|  | **LYs, RED-free** | **LYs, total** | **QALYs** | **Productive years lost**  **for patients** | **Productive years lost**  **for**  **caregivers** | **Events or recurrences** | **Active treatments for metastatic disease** | **Deaths** |
| --- | --- | --- | --- | --- | --- | --- | --- | --- |
| **Reference scenario** |  |  |  |  |  |  |  |  |
| Multi-cancer | 50,876 | 64,331 | 53,878 | 5,543 | 479 | 6,887 | 6,725 | 3,613 |
| RCC | 15,809 | 21,325 | 18,210 | 1,938 | 204 | 2,164 | 2,109 | 1,224 |
| Melanoma stage III | 5,509 | 8,543 | 7,375 | 1133 | 99 | 1,652 | 2,249 | 801 |
| TNBC | 22,823 | 25,396 | 20,431 | 1703 | 91 | 1,850 | 971 | 1043 |
| Melanoma stage II | 6,735 | 9,066 | 7,863 | 767 | 85 | 1,222 | 1,395 | 545 |
| **ESD scenario** |  |  |  |  |  |  |  |  |
| Multi-cancer | 56,431 | 66,886 | 56,443 | 4,747 | 374 | 5,166 | 4,943 | 2,733 |
| RCC | 17,496 | 22,019 | 18,843 | 1,679 | 171 | 1,764 | 1,802 | 990 |
| Melanoma stage III | 6,909 | 9,163 | 7,958 | 934 | 75 | 1,233 | 1,560 | 594 |
| TNBC | 24,328 | 26,282 | 21,236 | 1,366 | 63 | 1,246 | 598 | 719 |
| Melanoma stage II | 7,698 | 9,422 | 8,207 | 667 | 65 | 923 | 982 | 429 |
| **Change in outcomes  (% change)** |  |  |  |  |  |  |  |  |
| Multi-cancer | 5,555 (11%) | 2,556 (4%) | 2,365 (4.%) | -796(-14%) | -105 (-21%) | -1,722 (-25%) | -1,782 (-27%) | -881 (-24%) |
| RCC | 1,687 (11%) | 694 (3%) | 633 (3%) | 260(-13%) | 33(16%) | -400 (-18%) | -307 (-15%) | -234 (-19%) |
| Melanoma stage III | 1,400 (25%) | 620 (7%) | 582 (8%) | -199(-24%) | 24(-24%) | -419 (-25.0%) | -690 (-31%) | -207 (-26%) |
| TNBC | 1,505 (7%) | 886 (3%) | 805 (4%) | -237(-14 %) | -28(-31%) | -604 (-33%) | -373 (-38%) | -324 (-31%) |
| Melanoma stage II | 963 (14%) | 356 (4%) | 345 (4%) | -100 (-13%) | -20(-23%) | -174 (-15.1%) | -413 (-30.0%) | -116 (-21%) |

ESD, early-stage disease; LY, life-year; PD-1, programmed cell death protein 1; QALY, quality-adjusted life-year; RCC, renal cell carcinoma; RED, recurrence-/event-/disease-; TNBC, triple-negative breast cancer

### Appendix I

*Disaggregated Economic Results of the Analysis per indication.*

The table underneath shows the incremental impact per patient receiving treatment with Anti-PD(L)1’s in the ESD scenario in €.

|  | 2023 | 2024 | 2025 | 2026 | 2027 | 2028 | 2029 | 2030 | 2031 | 2032 | Average net impact per indication |
| --- | --- | --- | --- | --- | --- | --- | --- | --- | --- | --- | --- |
| TNBC | 28,563 | 38,972 | 55,385 | 52,625 | 54,850 | 54,584 | 50,034 | 47,027 | 44,216 | 42,360 | 46,862 |
| RCC | 33,654 | 54,183 | 49,598 | 48,136 | 39,763 | 37,358 | 37,875 | 36,570 | 36,535 | 38,927 | 41,260 |
| Melanoma Stage II | 30,532 | 48,455 | 39,837 | 30,236 | 18,998 | 9,909 | 2,723 | 2,623 | -4,236 | -9,664 | 16,941 |
| Melanoma Stage III | 18,831 | 24,259 | 10,898 | -2,837 | -14,673 | -25,770 | -33,873 | -40,873 | -42,560 | -50,041 | -15,664 |
| Weighted Average | 28,898 | 42,444 | 44,742 | 39,596 | 34,959 | 31,195 | 27,413 | 25,012 | 22,964 | 21,304 | 31,853 |

### Appendix J

According to the inputs of the model, over a 10-year time horizon, 12,894 patients are projected to receive adjuvant/neoadjuvant treatment with anti-PD-(L)1 agents out of a total of 16,596 eligible patients. This amounts to 78% of all eligible patients expected to receive active treatment with anti-PD(L)1 inhibitors throughout the 10-year time horizon.

Estimated target population and number of patients receiving anti-PD-(L)1 agents for early-stage disease, by year

|  | **2023** | **2024** | **2025** | **2026** | **2027** | **2028** | **2029** | **2030** | **2031** | **2032** | Cumulative |
| --- | --- | --- | --- | --- | --- | --- | --- | --- | --- | --- | --- |
| Patients receiving ESD anti-PD-(L)1^B^ | 907 | 1,233 | 1,242 | 1,267 | 1,303 | 1,388 | 1,390 | 1,388 | 1,388 | 1,388 | 12,894 |
| Patients in reference scenario | 745 | 427 | 411 | 388 | 354 | 276 | 275 | 275 | 275 | 276 | 3,702 |
| Target population ^A^ | 1,652 | 1,660 | 1,653 | 1,655 | 1,657 | 1,664 | 1,665 | 1,663 | 1,663 | 1,664 | 16,596 |

ESD, early-stage disease; PD-(L)1, programmed cell death protein 1 or its ligand A The estimated number of patients in Greece with melanoma (stage IIB-C and III), renal cell carcinoma, or triple-negative breast cancer who are eligible for early-stage cancer treatment with anti-PD-(L)1 agents. This is the target population used for both the reference and ESD scenarios. B The estimated number of patients from the target population who receive ESD treatment with anti-PD-(L)1 agents in the model under the ESD scenario.

| Patients receiving treatment with Anti-PD(L)1's in the ESD Scenario per indication | | | | | | | | | | | | |
| --- | --- | --- | --- | --- | --- | --- | --- | --- | --- | --- | --- | --- |
|  | 2023 | 2024 | 2025 | 2026 | 2027 | 2028 | 2029 | 2030 | 2031 | 2032 | Cumulative |  |
| TNBC | 195 | 517 | 529 | 548 | 554 | 2,772 | 554 | 554 | 554 | 554 | 5,116 |  |
| RCC | 331 | 335 | 330 | 330 | 358 | 2,187 | 413 | 440 | 476 | 476 | 3,872 |  |
| Stage III | 182 | 183 | 185 | 191 | 193 | 972 | 201 | 201 | 203 | 203 | 1,906 |  |
| Stage II | 198 | 198 | 198 | 198 | 198 | 1,011 | 194 | 194 | 194 | 194 | 2,001 |  |
| Cumulative | 906 | 1,233 | 1,242 | 1,267 | 1,303 | 1,334 | 1,362 | 1,389 | 1,427 | 1,431 | 12,894 |  |
